# Use Of POCUS for the Paediatric Patient with an Undifferentiated Upper Limb Injury

**DOI:** 10.24908/pocus.v8i1.15867

**Published:** 2023-04-26

**Authors:** David J McCreary, Alex White

**Affiliations:** 1 Paediatric Emergency Medicine Department, Sunderland Royal Hospital, South Tyneside and Sunderland NHS Foundation Trust United Kingdom

**Keywords:** POCUS, fracture, pulled elbow, nursemaid's elbow, joint effusion

## Abstract

A 2-year-old girl presented to the Paediatric Emergency Department following an unwitnessed injury to her left arm while playing at nursery limiting further examination. On assessment she was reluctant to use her left arm and further examination was difficult. In cases of unwitnessed and undifferentiated elbow injuries point of care ultrasound (POCUS) can be used to evaluate for elbow joint effusion, fracture, or radial head subluxation, also known as nursemaid’s elbow. Pulled elbow is a commonly encountered paediatric injury but based on the history and examination findings it may not always be obvious. We present an approach to the child with an undifferentiated elbow injury incorporating POCUS as a means of increasing the reliability of findings on clinical examination.

## Case

A 2-year-old girl presented to the Paediatric Emergency Department (PED) following an unwitnessed injury to her left arm while playing at nursery. The child had no significant past medical history. On presentation the child was in distress and holding her left arm adducted and in full flexion supported by her unaffected arm. Following the incident, she had not used the affected arm at all. On examination there was no evidence of swelling, deformity, or bruising. She was neurovascularly in-tact. At this time the diagnoses under consideration were bony injury compared with pulled elbow (nursemaid’s elbow).

**Figure 1:**
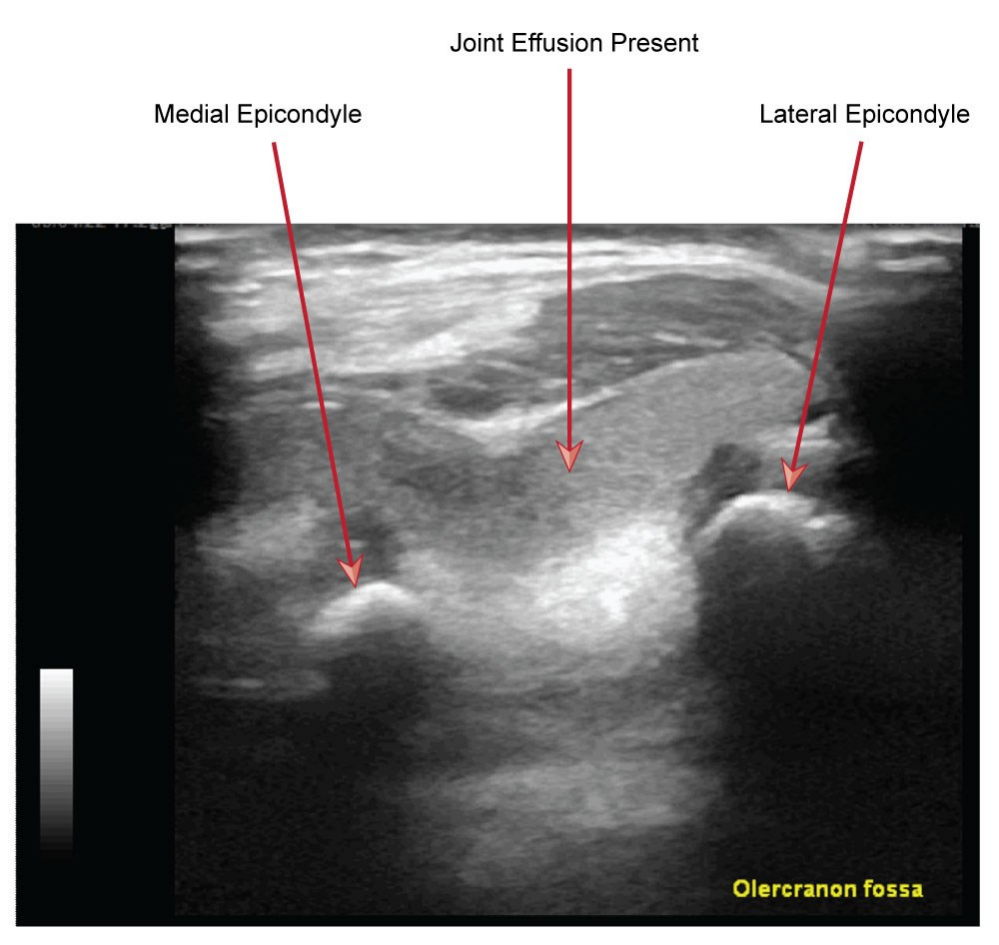
POCUS view of olecranon fossa of elbow in transverse plane: a moderate sized effusion is present causing the triceps muscle and tendon to elevate.

**Figure 2:**
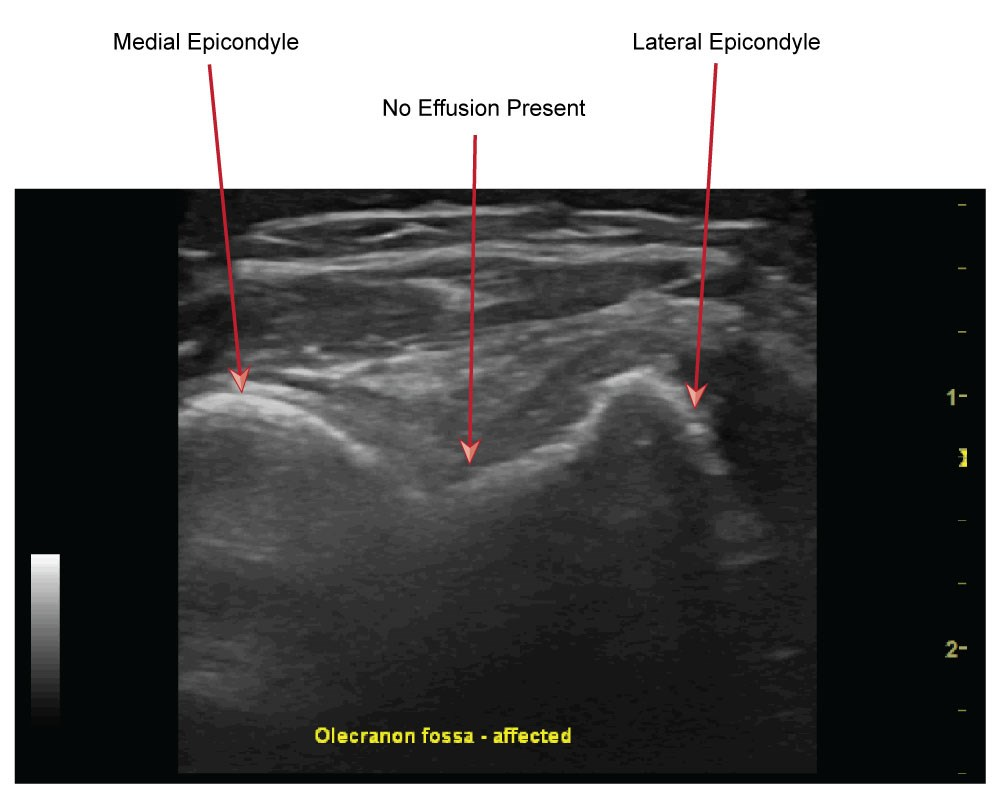
POCUS view of olecranon fossa of elbow in transverse plane, no effusion is present.

In our PED, POCUS is utilised by those who are appropriately trained and it has demonstrated a high degree of reliability in differentiating the above injuries. Our approach was as follows: an initial scan for the presence of a joint effusion which is suggestive of an intracapsular fracture (see Figures 1 & 2). Then an interrogation of common sites of occult bony injury, such as the radial head along with the distal humerus. Finally, when the above views did not provide an obvious site of injury, we undertook views to evaluate for signs of nursemaid’s elbow.

Using POCUS, the images we obtained of the elbow joint were without evidence of effusion in both the long and short axes at the olecranon fossa. There was no evidence of bony injury in the distal humerus or radial head. Interrogating along the radiocapitellar line to assess for features of nursemaid’s elbow, there was a clear visualization of supinator muscle curling over the radial head along with an exaggerated synovial fringe (hook sign). Findings were compared to the contralateral, unaffected side and were different. Given these findings we were able to diagnose a nursemaid's elbow. The child subsequently underwent successful reduction of the radial head subluxation and was discharged after she demonstrated an immediate return of normal use of the arm. 

## Discussion

Assessing a young child with an undifferentiated upper limb injury can be challenging for a number of reasons including an indistinct clinical history, subtle clinical signs and their inability to fully cooperate with structured examination. Furthermore, radiographic findings can be difficult to interpret and appreciate, leading to incomplete conclusions such as the presence of an abnormal fat pad. Use of POCUS by appropriately trained clinicians, as an extension of a clinical examination, can help to identify or confirm the site of injury and thereby direct x-rays, reducing both unnecessary radiation as well as the time in the emergency department for the child and carer(s). POCUS has been demonstrated to be both sensitive and specific for forearm fractures, supracondylar fractures, and proximal humerus fractures when used by those following short periods of focussed training 1, 2. It is viewed as an extension of clinical examination and does not replace other investigations such as x-rays, which should be utilised as needed after POCUS assessment.

Nursemaid's Elbow is a radial head subluxation caused by axial traction on the extended arm while the forearm is pronated causing the radial head to slip underneath the annular ligament 3. This is a commonly encountered upper limb problem in young children and one that can be difficult to clinically diagnose using history and examination alone. Attempted reduction of suspected pulled elbow can occasionally be carried out when fracture is present leading to unnecessary distress and pain for the child 4. POCUS findings have been demonstrated to have 100% sensitivity and over 90% specificity in patients with nursemaid’s elbow and can be utilised in all cases in which the history isn’t clearly suggestive before reduction is considered 5.

## Technique

**Figure 3:**
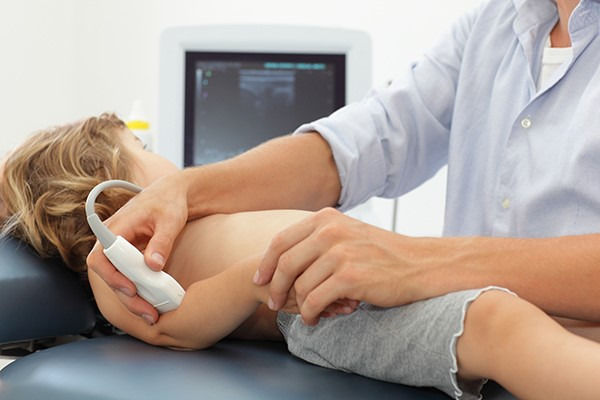
Transducer position to obtain views to confirm pulled elbow. In this image the ultrasound probe is orientated caudally.

In order to perform this scan, place the high frequency linear probe in long section at the level just above the radial head as shown in Figure 3.

**Figure 4:**
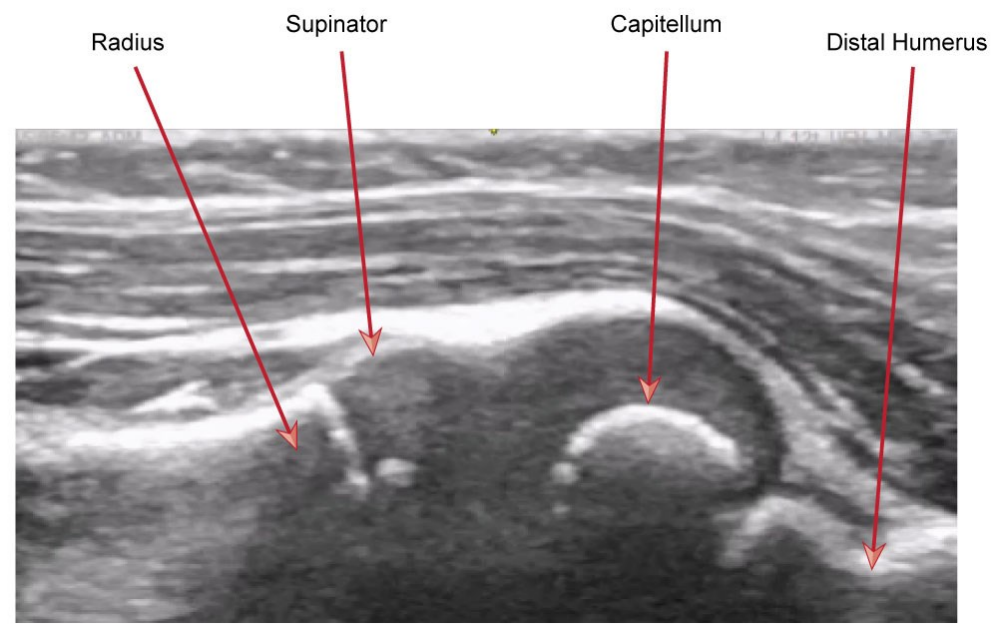
Sonographic anatomy – normal appearances. In this image the ultrasound probe is orientated caudally.

Position the probe along the radiocapitellar line so that the field of view includes the capitellum of the humerus and the area of capsule overlying it. The normal appearances are outlined in Figure 4.

When nursemaid’s elbow has occurred, there are two signs to be aware of: 

Firstly, the supinator muscle extends further than expected into the joint space with a hooked appearance proximally. This occurs because the supinator muscle has its origin at the annular ligament among other places. As the radial head moves relatively distally in pulled elbow this allows space for a portion of the supinator muscle to move proximally and inferiorly due a reduced tenting effect. This is known as the “hook sign”. In contrast in normal appearances, the supinator muscle tapers off to a straight point proximally. 

**Figure 5:**
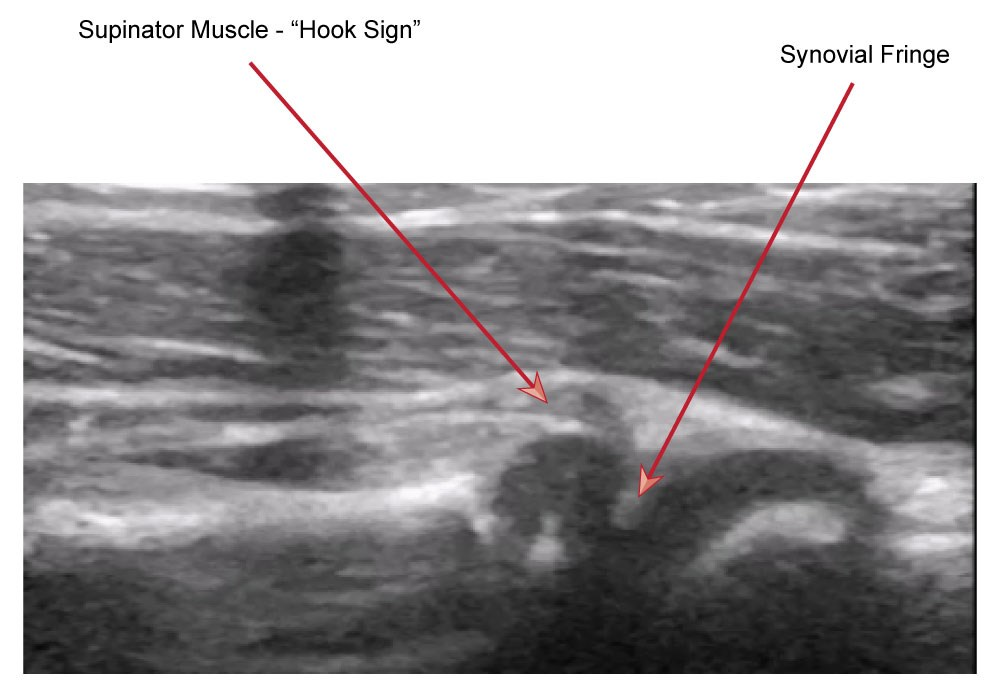
The sonographic signs consistent with pulled elbow.

Secondly, there is an increased prominence of the synovial fringe which appears to fall into the joint space and look more pointed. This is also thought to occur due to a tenting effect distally allowing the proximal portion to fall into a deeper position. These signs are highlighted in Figure 5. 

Both signs should be contrasted against the appearances of the unaffected elbow. If the signs are present and the remainder of the examination and clinical history are in keeping with nursemaid’s elbow, this would be a reasonable indication to attempt reduction. 

X-rays have no role in confirming nursemaid’s elbow or resolution following manipulation, but POCUS can be used to confirm that the reduction has successfully occurred. To confirm this re-scan the side which has undergone reduction in the original window and the hook sign and synovial appearances should have resolved. 

## Statement of consent

Consent has been granted from those with parental responsibility for the images this report.

## Conflicts of Interest

The authors have no conflicts of interest to declare. 
